# User Profiles of Private Long-term Care Services Not Fully Covered by Public Insurance in Japan

**DOI:** 10.31662/jmaj.2024-0164

**Published:** 2024-11-11

**Authors:** Kazuhiro Abe, Hiroshi Murayama

**Affiliations:** 1 Department of International Cooperation for Medical Education, International Research Center for Medical Education, Graduate School of Medicine, The University of Tokyo, Tokyo, Japan; 2 Division of Data-based Health Management, Health Innovation and Technology Center, Faculty of Health Sciences, Hokkaido University, Sapporo, Japan; 3 Research Team for Social Participation and Healthy Aging, Tokyo Metropolitan Institute for Geriatrics and Gerontology, Tokyo, Japan

**Keywords:** aged, long-term care, home care services, private, Japan

## Abstract

**Introduction:**

This study aimed to evaluate the characteristics of private long-term care (LTC) service users provided by a company independent from public LTC insurance (LTCI) and to analyze the usage patterns across different types of services.

**Methods:**

We utilized data from 8,046 consultations from the administration data of a private LTC service in Suginami Ward, Tokyo, Japan. We focused on older adults enrolled from February 2016 to October 2019 with follow-up until June 2020. The descriptions included users’ demographics, LTCI-certified care levels, living situations, and reasons for choosing private LTC services. Furthermore, we examined the frequencies and minutes of each type of service used, such as shopping, meal, cleaning, outing, and social participation assistance, stratified by solitary living and LTCI certification.

**Results:**

The study included 51 older adults, including 35 (69%) women, 28 (55%) solitary living individuals, 23 (45%) public LTCI-certified individuals, and 45 (88%) participants residing in detached houses. The primary motive for private service use was the absence of informal caregiving in 55% of the participants. Cleaning assistance was the most frequently used. Solitary living residents used various types of assistance, not only cleaning, and LTCI-certified individuals more frequently used meal and outing assistance than those without LTCI certification.

**Conclusions:**

These findings indicate that older adults using private LTC services predominantly lived alone, lived in detached houses, or had no informal care support. Our findings provide an opportunity to examine the appropriateness of the complementary relationship between public and private LTC services.

## Introduction

The Japanese government has maintained a compulsory public long-term care insurance (LTCI) system since 2000 to cope with the demands of the aging society ^(1)^. This LTCI allows people aged 65 years and above with certified LTC needs and those aged 40 years and above with intractable diseases to receive LTC services at home, day-care centers, and facilities. Nevertheless, private LTC services are offered independently of public LTCI ^(2)^. Private LTC companies usually provide assistance to older adults who are ineligible for the public LTCI system or provide support not covered by the public LTCI to older people regardless of their LTCI eligibility ^(2)^. To answer the diverse needs of older people and ensure the sustainability of the LTCI system, the government also recommends that older people use a combination of private LTC services and LTC services covered by the public LTCI ^(3), (4), (5), (6), (7), (8), (9)^. To determine whether older Japanese adults have reasonable access to LTC services through both public and private providers, it is necessary to know the characteristics of users and their utilization patterns. Although the user profiles and utilization patterns of public LTC services can be identified by analyzing LTCI claims data ^(10)^, the utilization of private LTC services is challenging due to a lack of available data.

A survey involving private LTC providers in Japan has previously been published ^(2)^. Although this survey provided an overview of each private service, it failed to report the characteristics of the users and patterns of services used. Furthermore, the usage patterns of private LTC services may considerably vary depending on the user’s living environment and public LTCI eligibility. These factors should be considered.

Therefore, the present study aimed to describe the characteristics of users and the trend of services used by the types of services. Customer data from a private LTC service provider in Tokyo, Japan, were used. The collected data were stratified by solitary living and eligibility for public LTCI.

## Materials and Methods

### Study design, data, participants, and settings

We retrieved the administrative data for the My Home Concierge service provided by SECOM Living Partner Kugayama (SECOM. Co., Ltd.) in Suginami Ward, Tokyo metropolitan area. The study cohort comprised 51 older adults who had signed up for the My Home Concierge service. Those who signed up for the service from February 2016 to October 2019 were followed up until June 2020. Data on 8,046 consultations were collected.

The My Home Concierge service provides 24-h consultation for problems in daily life, reasonable solutions, and assistance in resolving them to subscribers ^(11)^. For example, users can ask its staff to do daily shopping on their behalf, accompany them to the hospital, clean their rooms, or care for their gardens. During the study period, the monthly fee, covering 3 h of service per month, was 18,000 yen. Individuals who wanted to use the service for more than 3 h per month paid an additional fee of 4,000 yen per 0.5 h.

### Trends of services used

The study outcomes were the monthly averages of frequencies and minutes of services used, which were calculated by dividing the numbers and minutes of services used by the number of contracted days, multiplied by 30. If the service time was less than 30 min, the time was recorded as 30 min. If the service time was longer than 30 min, the time was recorded in 15-min increments after 30 min.

The frequencies and minutes of services used were categorized by the type of service: shopping assistance, meal assistance, cleaning assistance (e.g., daily cleaning), special cleaning and yard work (e.g., cleaning of air conditioners), facility management assistance (e.g., repair of buildings and furniture), digital support (e.g., use of the Internet), outing assistance (e.g., arrangement of transportation like a taxi), medical assistance (e.g., hospital visits and medication management), LTCI assistance (e.g., paperwork for public LTCI), money management assistance (e.g., asset management, insurance, taxes, and legal procedures), social participation assistance (e.g., assistance for travel and hobbies), and social contact (e.g., conversation). Table 1 presents the types of services and their contents in the My Home Concierge service. Special cleaning and yard work, facility management assistance, digital support, money management assistance, and social participation assistance are not covered by the public LTCI.

**Table 1.** Service Types and Contents.

Type of service	Content of services for older people
Shopping assistance	Shopping on behalf of older people.
Meal support	Preparing meals, cleaning up dishes, as well as providing information on and applying for meal delivery services.
Cleaning assistance	Providing daily cleaning, laundry, and garbage disposal services.
Special cleaning and yard work assistance^†^	Providing special services, such as cleaning of exhaust fans and air conditioners, disposing of bulky waste, and yard work.
Facility management support^†^	Furniture repair and installation, redecoration, building repair and maintenance, as well as disaster and crime prevention.
Digital support^†^	Assisting with the use of the Internet, cell phones, computers, and appliances.
Outing assistance	Assisting when going out and arranging transportation such as cabs.
Medical assistance	Assisting with medical visits, hospitalization, discharge, and medication management as well as providing home visit services.
Long-term care insurance assistance	Assisting with procedures for public long-term care services such as day services, short-stay services, and renting long-term care equipment.
Money management assistance^†^	Assisting with asset management, insurance, taxes, and legal procedures.
Social participation support^†^	Providing information and assisting with procedures related to travel and hobbies.
Social contact	Providing conversation.

^†^ Not covered by long-term care services based on public long-term care insurance in Japan.

### Participants’ characteristics

To better understand the characteristics of private service users, we analyzed the participants’ age, sex, LTCI-certified care levels, household structure (i.e., living alone, living alone but family members live in the same building/on the same property [two-household house], living with a spouse, living with children, living with grandchildren, or living with a spouse and grandchildren), relatives living nearby, housing type (i.e., detached house or apartment building), marital history (i.e., unmarried, married [living] or married [bereaved]), and reason for using this private service (i.e., bereavement and living alone, lack of informal caregiving, diseases/aging/cognitive decline, or not covered by LTCI). The care levels for Japan’s LTCI include two stages that require support for instrumental activities of daily living (support levels 1 and 2) and five that require LTC for activities of daily living (ADL; care levels 1-5, a higher level indicates greater need for care) ^(12)^.

### Statistical analysis

The numbers and proportions of participation during the first year of the service contract were stratified by solitary living and LTCI certification. As the monthly frequencies and minutes of each service use were not normally distributed, median with interquartile range (IQR) is reported. The data are plotted using box plots to demonstrate their distributions. Wilcoxon’s rank-sum test was employed to compare the two groups. For users with continuous contracts for more than 2 years, scatter plots and quadratic predictions with 95% confidence intervals were used to observe the trends in monthly mean frequencies and the minutes of each service use during the first 2 years of the contract.

All data management and analyses were conducted using Stata 16 MP (College Station, TX; StataCorp LLC.). A *P*-value <0.05 was considered to be statistically significant.

## Results

Data from 51 older adults, including 28 (55%) solitary living individuals and 23 (45%) public LTCI-certified individuals, were analyzed (Table 2). Of the participants, 35 (69%) were women, of whom 23 (82%) were living alone and 16 (70%) were certified by the LTCI. The participants’ median (IQR) age was 84 (81-88) years. LTCI-certified older adults belonged to support level 1 (n = 11, 48%), support level 2 (n = 4, 17%), or care level 1 (n = 8, 35%). In total, 45 (88%) participants resided in detached houses, whereas the remaining lived in apartment houses. The common reasons for using private LTC services were a lack of informal caregiving (55%), followed by diseases/aging/cognitive decline (45%). Among the participants, 34 (77%) continued to use the My Home Concierge service for over 2 years and 17 (33%) for over 3 years.

**Table 2.** Baseline Characteristics of Users Stratified by Solitary Living and Long-Term Care Insurance Certification.

	Total (n = 51), n (%)	Solitary living, n (%)		Long-term care certification, n (%)	
		Solitary (n = 28)	Nonsolitary (n = 23)	Certified (n = 23)	Not certified (n = 28)
Sex					
Male	16 (31%)	5 (18%)	11 (48%)	7 (30%)	9 (32%)
Female	35 (69%)	23 (82%)	12 (52%)	16 (70%)	19 (68%)
Age, median (interquartile range)	84.0 (81.0-88.0)	83.5 (80.0-88.0)	84.0 (81.0-89.0)	84.0 (81.0-89.0)	82.5 (79.5-87.0)
Care level					
Self-reliance or unapplied	28 (55%)	14 (50%)	14 (61%)	0 (0%)	28 (100%)
Support level 1	11 (22%)	7 (25%)	4 (17%)	11 (48%)	0 (0%)
Support level 2	4 (8%)	1 (4%)	3 (13%)	4 (17%)	0 (0%)
Care level 1	8 (16%)	6 (21%)	2 (9%)	8 (35%)	0 (0%)
Household structure					
Solitary living	28 (55%)	28 (100%)	0 (0%)	14 (61%)	14 (50%)
Living alone but family members live in the same building (two-household house)	6 (12%)	0 (0%)	6 (26%)	3 (13%)	3 (11%)
Living with a spouse	11 (22%)	0 (0%)	11 (48%)	3 (13%)	8 (29%)
Living with children	3 (6%)	0 (0%)	3 (13%)	2 (9%)	1 (4%)
Living with grandchildren	2 (4%)	0 (0%)	2 (9%)	1 (4%)	1 (4%)
Living with a spouse and grandchildren	1 (2%)	0 (0%)	1 (4%)	0 (0%)	1 (4%)
Relatives living nearby					
Yes	16 (31%)	5 (18%)	11 (48%)	8 (35%)	8 (29%)
Housing type					
Detached house	45 (88%)	23 (82%)	22 (96%)	21 (91%)	24 (86%)
Apartment house	6 (12%)	5 (18%)	1 (4%)	2 (9%)	4 (14%)
Marital history					
Unmarried	4 (8%)	3 (11%)	1 (4%)	1 (4%)	3 (11%)
Married (living)	16 (31%)	3 (11%)	13 (57%)	5 (22%)	11 (39%)
Married (bereaved)	31 (61%)	22 (79%)	9 (39%)	17 (74%)	14 (50%)
Reason for using the service^†^					
Lack of informal caregiving	28 (55%)	15 (54%)	13 (57%)	12 (52%)	16 (57%)
Diseases, aging, or cognitive decline	23 (45%)	12 (43%)	11 (48%)	14 (61%)	9 (32%)
Bereavement and living alone	11 (22%)	10 (36%)	1 (4%)	4 (17%)	7 (25%)
Not covered by long-term care insurance	5 (10%)	4 (14%)	1 (4%)	2 (9%)	3 (11%)

^†^ Multiple answers allowed.

As presented in Table 3, the service was used 8.38 (4.60-13.23) times and for 216.62 (176.01-279.93) min per month. The average proportion of individuals who used the service for more than 180 min per month, for which they paid additional fees, was 66.6% per month in the first year and 54.0% in the second year. Cleaning assistance and special cleaning, including yard work, were the most common and time-consuming services requested. Cleaning assistance was used 1.64 (0.49-2.55) times and for 55.51 (18.00-127.25) min as the median (IQR) of monthly personal means. Special cleaning, including yard work, was provided 1.50 (0.66-2.79) times and for 56.50 (24.25-105.64) min. Figure S1 and S2 present the distributions of frequencies and minutes of each service used as box plots and outliers.

**Table 3.** Monthly Median Values of Frequency and Minutes of Each Service Stratified by Solitary Living.

		Frequency				Minutes		
	Total, median (IQR^†^)	Nonsolitary, median (IQR^†^) (n = 23)	Solitary, median (IQR^†^) (n = 28）	*P*-value^‡^	Total, median (IQR^†^)	Nonsolitary, median (IQR^†^) (n = 23)	Solitary, median (IQR^†^) (n = 28）	*P*-value^‡^
Total number of times of service use	8.38 (4.60-13.23)	7.81 (5.51-11.01)	9.70 (4.40-15.87)	0.30	216.62 (176.01-279.93)	220.77 (181.28-276.74)	209.38 (161.76-292.27)	0.72
Shopping assistance	0.16 (0-0.58)	0.08 (0-0.25)	0.25 (0.08-1.64)	0.012	3.70 (0-20.98)	3.08 (0-5.60)	5.00 (1.15-27.85)	0.051
Meal support	0 (0-0.16)	0 (0-0)	0 (0-0.16)	0.15	0 (0-0.97)	0 (0-0)	0 (0-4.07)	0.18
Cleaning assistance	1.64 (0.49-2.55)	1.89 (1.23-2.96)	0.82 (0.33-2.47)	0.066	55.51 (16.08-119.42)	101.79 (47.20-133.16)	27.87 (5.91-71.77)	0.009
Special cleaning and yard work assistance^§^	1.50 (0.66-2.79)	1.89 (1.07-2.79)	1.48 (0.45-2.59)	0.35	56.50 (24.25-105.64)	56.50 (31.36-113.28)	58.33 (15.81-91.76)	0.32
Facility management support^§^	0.58 (0.25-1.23)	0.25 (0.16-1.23)	0.70 (0.38-1.27)	0.25	10.60 (5.18-24.45)	10.01 (2.88-24.27)	10.79 (7.79-25.54)	0.28
Digital support^§^	0.16 (0.08-0.82)	0.16 (0.08-1.23)	0.25 (0.08-0.70)	0.55	6.08 (0.41-19.93)	6.16 (1.23-31.64)	6.07 (0.08-14.99)	0.30
Outing assistance	0.08 (0-0.33)	0.08 (0-0.33)	0.08 (0-0.33)	0.88	0.16 (0-10.19)	0 (0-11.98)	0.33 (0-8.94)	0.72
Medical service assistance	0.08 (0-1.40)	0.08 (0-1.64)	0.25 (0-1.23)	0.58	1.64 (0-19.18)	0.22 (0-19.18)	2.90 (0-20.76)	0.37
Long-term care service assistance	0 (0-0.16)	0 (0-0)	0 (0-0.25)	0.038	0 (0-3.16)	0 (0-0)	0 (0-5.03)	0.016
Money management assistance^§^	0 (0-0.25)	0 (0-0.08)	0 (0-0.37)	0.14	0 (0-4.93)	0 (0-1.07)	0 (0-6.78)	0.16
Social participation support^§^	0 (0-0.08)	0 (0-0.25)	0 (0-0)	0.051	0 (0-0)	0 (0-0.89)	0 (0-0)	0.072
Social contact	0.58 (0.33-1.64)	0.49 (0.33-0.90)	0.62 (0.34-2.05)	0.24	2.38 (0.58-12.90)	0.99 (0.59-3.94)	6.26 (0.54-29.15)	0.094

^†^ IQR: interquartile range ^‡^ Wilcoxon’s rank-sum test ^§^ Not insured by public long-term care insurance

Some types of services were used differently by solitary living participants and those with LTCI certification, as shown in Table 3 and 4. Solitary living individuals used more shopping and LTC assistance than nonsolitary living ones (median [IQR] of nonsolitary vs. solitary: 0.08 (0-0.25) vs. 0.25 [0.08-1.64] for the frequency of shopping assistance, 3.08 [0-5.60] vs. 5.00 [1.15-27.85] for the minutes of shopping assistance, 0 (0-0) vs. 0 [0-0.25] for the frequency of LTC assistance, and 0 (0-0) vs. 0 (0-5.03) for the minutes of LTC assistance. However, solitary persons used fewer minutes of cleaning assistance than nonsolitary ones: 101.79 (47.20-133.16) vs. 27.87 (5.91-71.77). LTCI-certified individuals more frequently used meal, facility management, outing, medical, and money management assistance than those without LTCI certification (median [IQR] of uninsured vs. insured, 0 [0-0] vs. 0 [0-0.25] for meal assistance, 0.38 [0.21-0.82] vs. 1.07 [0.49-1.48] for facility management assistance, 0 [0-0.16] vs. 0.25 [0-1.15] for outing assistance, 0.08 [0-0.37] vs. 0.74 [0.08-2.47] for medical assistance, and 0 [0-0] vs. 0.25 [0-0.58] for money management assistance. In addition, LTCI-certified individuals spent more time with medical and money management assistance from the private LTC service than those without LTCI certification: 0.18 (0-4.88) vs. 15.12 (0.62-25.48) for medical assistance and 0 (0-0) vs. 0.79 (0-6.95) for money management assistance.

**Table 4.** Monthly Median Values of Frequency and Minutes of Each Service Use Stratified by Long-Term Care Certification.

	Frequency				Minutes			
	Total, median (IQR^†^)	Not certified, median (IQR^†^) (n = 28)	Certified, median (IQR^†^) (n = 23）	*P*-value^‡^	Total, median (IQR^†^)	Not certified, median (IQR^†^) (n = 28)	Certified, median (IQR^†^) (n = 23）	*P*-value^‡^
Total number of times of service use	8.38 (4.60-13.23)	6.70 (3.78-11.01)	11.26 (6.16-17.18)	0.006	216.62 (176.01-279.93)	197.10 (149.42-266.27)	226.75 (189.91-304.60)	0.12
Shopping assistance	0.16 (0-0.58)	0.08 (0-0.25)	0.25 (0-1.15)	0.16	3.70 (0-20.98)	3.02 (0-5.13)	5.92 (0-27.99)	0.22
Meal support	0 (0-0.16)	0 (0-0)	0 (0-0.25)	0.048	0 (0-0.97)	0 (0-0)	0 (0-4.68)	0.062
Cleaning assistance	1.64 (0.49-2.55)	1.56 (0.60-3.16)	1.73 (0.49-2.38)	0.61	55.51 (16.08-119.42)	59.30 (18.00-127.25)	55.51 (6.88-109.52)	0.53
Special cleaning and yard work assistance^§^	1.50 (0.66-2.79)	1.61 (0.86-2.51)	1.48 (0.49-3.37)	0.97	56.50 (24.25-105.64)	62.14 (39.42-108.87)	31.56 (16.08-98.19)	0.23
Facility management support^§^	0.58 (0.25-1.23)	0.38 (0.21-0.82)	1.07 (0.49-1.48)	0.020	10.60 (5.18-24.45)	10.06 (3.09-25.54)	14.94 (7.56-24.27)	0.31
Digital support^§^	0.16 (0.08-0.82)	0.25 (0.04-0.82)	0.16 (0.08-0.82)	0.63	6.08 (0.41-19.93)	4.32 (0.32-20.55)	6.58 (0.41-14.75)	0.80
Outing assistance	0.08 (0-0.33)	0 (0-0.16)	0.25 (0-1.15)	0.012	0.16 (0-10.19)	0 (0-6.05)	1.23 (0-16.19)	0.057
Medical service assistance	0.08 (0-1.40)	0.08 (0-0.37)	0.74 (0.08-2.47)	0.008	1.64 (0-19.18)	0.18 (0-4.88)	15.12 (0.62-25.48)	0.023
Long-term care service assistance	0 (0-0.16)	0 (0-0.04)	0 (0-0.49)	0.051	0 (0-3.16)	0 (0-0)	0 (0-7.59)	0.055
Money management assistance^§^	0 (0-0.25)	0 (0-0)	0.25 (0-0.58)	0.008	0 (0-4.93)	0 (0-0)	0.79 (0-6.95)	0.017
Social participation support^§^	0 (0-0.08)	0 (0-0.04)	0 (0-0.08)	0.95	0 (0-0)	0 (0-0.04)	0 (0-0)	0.61
Social contact	0.58 (0.33-1.64)	0.53 (0.33-1.11)	0.58 (0.33-2.55)	0.40	2.38 (0.58-12.90)	1.78 (0.39-9.41)	3.94 (0.59-28.19)	0.15

^†^ IQR: interquartile range ^‡^ Wilcoxon’s rank-sum test ^§^ Not insured by public long-term care insurance

**Figure 1.**
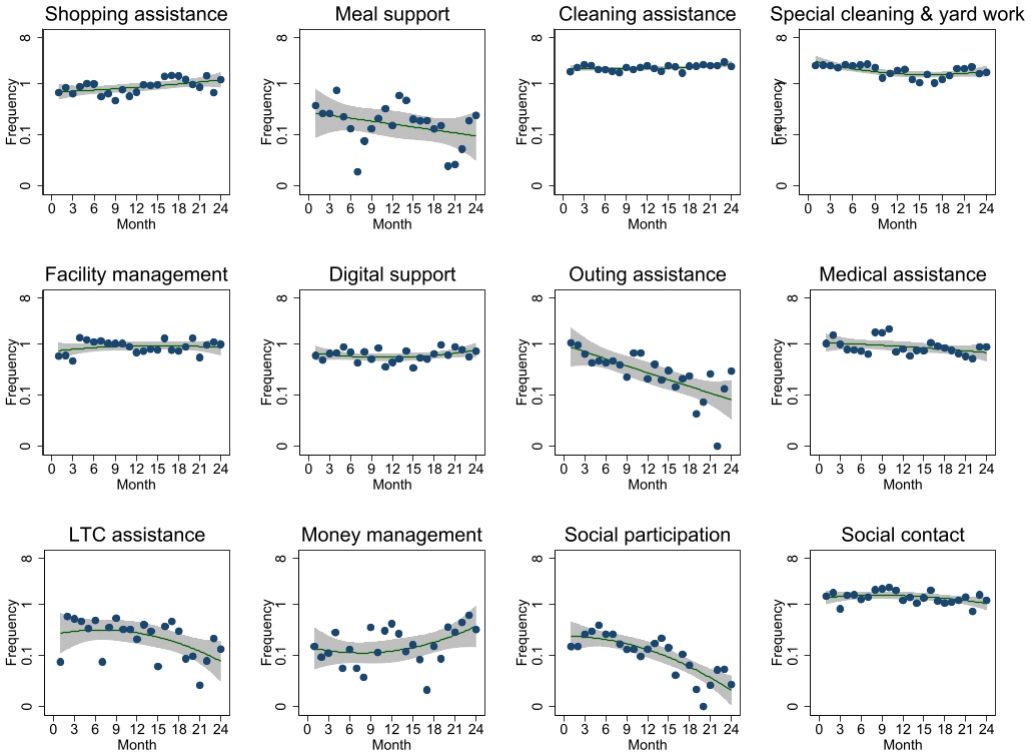
Trends of the monthly mean frequency of each service use during the first 2 years of the contract. Scatterplots and quadratic prediction plots with 95% confidence intervals are shown.

Figure 1 and S3 show that most types of services were consistently used. However, the frequencies and minutes used for outing and social participation assistance gradually decreased over the first 2 years of the contract.

## Discussion

This study shows the characteristics of private LTC service users as well as the frequency and minutes of each type of service used stratified by solitary living and public LTCI certification. The results indicated that 55% of users lived alone and 88% resided in detached houses. The most common reason for using the private service was the lack of informal caregiving, followed by diseases/aging/cognitive decline. The users mainly requested cleaning assistance, including yard work. Solitary older adults needed more shopping and LTCI assistance than nonsolitary ones, whereas solitary ones used less cleaning assistance than nonsolitary ones. Services such as meal, facility management, outing, medical, and money management assistance were more frequently used by public LTCI-certified participants than those without certification. The frequencies and minutes of most types of services used remained stable, except for outing and social participation assistance.

According to a survey conducted by Japan’s Cabinet Office on citizens aged 60 years and above, 65.2% lived in detached houses in large cities, including Tokyo ^(13)^. In addition, 13.3% of men and 21.1% of women aged 65 years and above in Japan lived alone as of 2015 ^(13)^. Compared with these results, the proportions of our study participants living in detached houses and those living alone were considerably higher. These data suggest that the My Home Concierge service may be selected by older persons who live alone, have little family support, have difficulty maintaining a detached house, and can afford to pay for the service.

Private LTC service users highly demanded cleaning and special cleaning assistance with yard work. In a study examining the impact of the government’s 2006 reduction of the public LTCI service coverage for older adults with support levels, cleaning assistance was the most common decrease in the content of in-home care services after the coverage change ^(14)^. Furthermore, 11.1% of older adults who experienced such a decrease requested private LTC services no longer provided by in-home care at their own expense. Our results indicated that the demand for cleaning assistance among older adults has remained high since 2006. In addition, we found that private LTC services were used for routine cleaning assistance despite the use of insured services. Contrary to special cleaning, including yard work, cleaning assistance for areas of daily living is included in the insured services. Therefore, it is likely that routine cleaning by insured in-home care services is insufficient or that older adults do not use much of the space in their homes.

The higher use of shopping and LTCI assistance among solitary persons may have been caused by the lack of informal caregivers or the higher care levels of solitary participants. It was also unexpected that although solitary residents used most service types for longer periods than nonsolitary residents, they spent less time using cleaning assistance than the latter, possibly because this service is capped at 180 min monthly. This may have reduced the amount of time for cleaning as solitary residents spend more time on services other than cleaning, such as social contact. In other words, solitary older adults may need more than 180 min of assistance per month. It may also be possible that the proportion of participants living in apartments is higher in solitary than in nonsolitary residents, which means solitary residents may spend less time cleaning than nonsolitary residents living mainly in detached houses. In addition, solitary residents tend to be more housebound and may be less likely to feel the need to clean a home they are accustomed to for a long time ^(8)^.

The more frequent use of meal, facility management, outing, medical, and money management assistance among participants insured by the public LTCI compared with that among uninsured persons seems reasonable, considering the differences in the degree of care needed. However, whether insured and uninsured services are appropriately distinguished is necessary as meal and outing assistance can also be provided by insured in-home care services. For example, LTCI-provided meal assistance involves the preparation of meals for the insured person but not for the spouse or family members ^(15)^. Those who find this inconvenient may use private services.

Various reasons may have contributed to the gradual decrease in the frequency of use of outing and social participation assistance by the older adults over 2 years. As a premise, the company’s staff never refused service provision for outing and social participation assistance during the study period. First, the condition of an older person may decline, making it impossible for them to go out and participate in society (e.g., worsening of dementia, decline in ADL). Second, the private service introduces external services, such as travel agencies and day services, to older adults. In addition, in April 2017, the Japanese government introduced lifestyle support coordinators who coordinate resources such as community volunteers, nonprofit organizations, and private companies along with public LTC services in each municipality (i.e., comprehensive projects known as *Sogo Jigyo* in Japanese) ^(16)^. Thus, relationships between users and these services may have been established, reducing the need for outing and social participation assistance. Third, the last few months may have been affected by the COVID-19 pandemic. In April 2020, the Japanese government declared a state of emergency and recommended that people refrain from going out and participating in social activities ^(17)^. This could explain the decrease in the frequency of use of those services in the last few months.

### Limitations

This study has several limitations. First, the number of participants was small, restricting further analyses considering the heterogeneity of the population and the overlap of public and private LTC services. Therefore, the association of frequency and minutes of service use by solitary living and LTCI certification could not be determined due to possible confounders, such as the economic status of users. Second, the 2-year scatter plots were restricted to users under contract for over 2 years. This might have biased data toward users whose conditions were relatively stable. Third, we used the customer data of a company providing private LTC services around Suginami Ward, Tokyo. Therefore, the results of this study may not necessarily be consistent with the status of services in other regions or companies. Nevertheless, data on the use of private LTC services are largely inaccessible, and SECOM has provided security services to 2.57 million homes as of March 2024 (the highest number in Japan); therefore, our study results provide valuable data on the use of private LTC services by older adults ^(18)^.

### Conclusions and implications

This study found that the private LTC services in Tokyo were used by older adults living alone, living in detached houses, or lacking informal care. Independently of the additional use of LTCI-provided services, cleaning assistance was most frequently used in private services. Solitary older adults used various types of assistance more frequently than nonsolitary individuals, including but not restricted to cleaning. Furthermore, LTCI-certified older adults used more meal and outing assistance of the private service than those without certification. Thus, private LTC services complemented public LTC services. Our findings can be used to examine the coverage and quality of public LTC services. For example, solitary living may need to be considered when certifying the care levels in public LTCI. In addition, the usability of meal, outing, and cleaning assistance in public services may need to be improved. It would be necessary to gather feedback regarding public LTC services from users and make efforts to continuously improve the quality and usability of public services considering the limited resources and service priorities. The results can also be used by private LTC providers to determine the demand from older people requiring less assistance. The contents of public and private LTC services should be balanced to ensure that older adults experience humane and fulfilled lives until the end.

## Article Information

### Conflicts of Interest

None

### Sources of Funding

This work was supported by the KAKENHI grant 19K22745 (to Dr. Murayama) from the Japan Society for the Promotion of Science.

### Acknowledgement

We thank the staff of SECOM Living Partner Kugayama for their contributions in providing data and advice on the interpretation of the results.

### Author Contributions

All authors contributed to study concept and design as well as the interpretation of data. In addition, Kazuhiro Abe drafted the manuscript and performed statistical analyses. Hiroshi Murayama takes responsibility for the acquisition of data, critical revision of the manuscript, funding acquisition, administrative support, and supervision. All authors have agreed to the final submitted manuscript.

### ORCID iD

Hiroshi Murayama: 0000-0003-2991-7763

### Approval by Institutional Review Board (IRB)

The protocol of this study was approved by the Research Ethics Committee of the University of Tokyo (No. 19-24) on May 8, 2019.

### Informed Consent

The requirement for informed consent was waived by the Research Ethics Committee because deidentified data were used.

### Data Availability Statement

The analyzed dataset was provided by SECOM Living Partner Kugayama, which does not permit to share it with third parties.

## Supplement

Supplementary Material
