# Supplementary material for: User Profiles of Private Long-term Care Services Not Fully Covered by Public Insurance in Japan
Source: JMA J. 2024 Nov 11;8(1):165–73. doi: 10.31662/jmaj.2024-0164 (PMC11799566; doi:10.31662/jmaj.2024-0164)
Supplement: Supplementary Material [file 2433-3298-8-1-0165-s001.pdf]

Supplemental materials

Figure S1. Monthly mean frequencies of each service used during the first year of the contract.
Figure S2. Monthly mean minutes of each service used during the first year of the contract.
Figure S3. Trends in monthly mean minutes of each service used during the first two years of the contract.

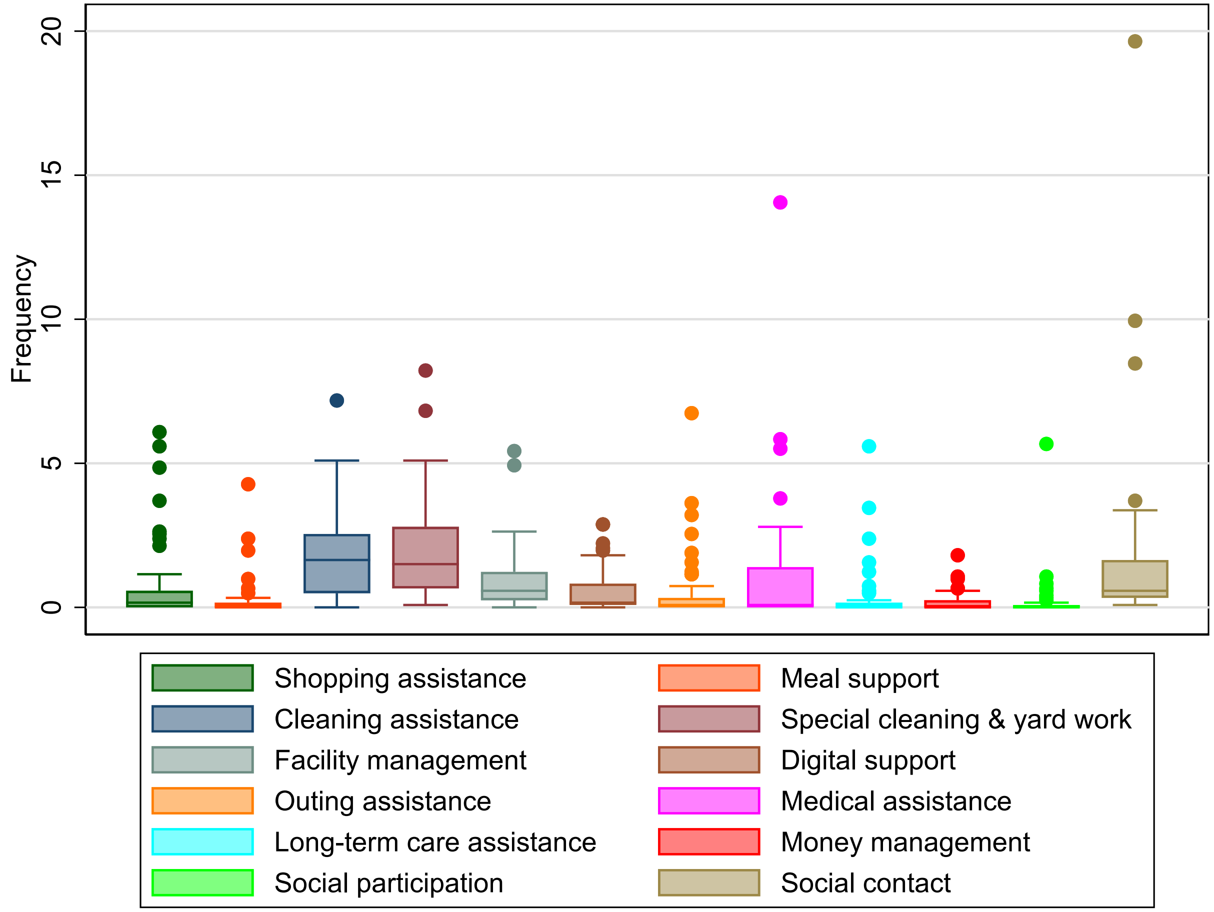

Figure S1 Monthly mean frequencies of each service used during the first year of the contract. The monthly frequency was calculated by dividing the number of services used by the number of contracted days, multiplied by 30.

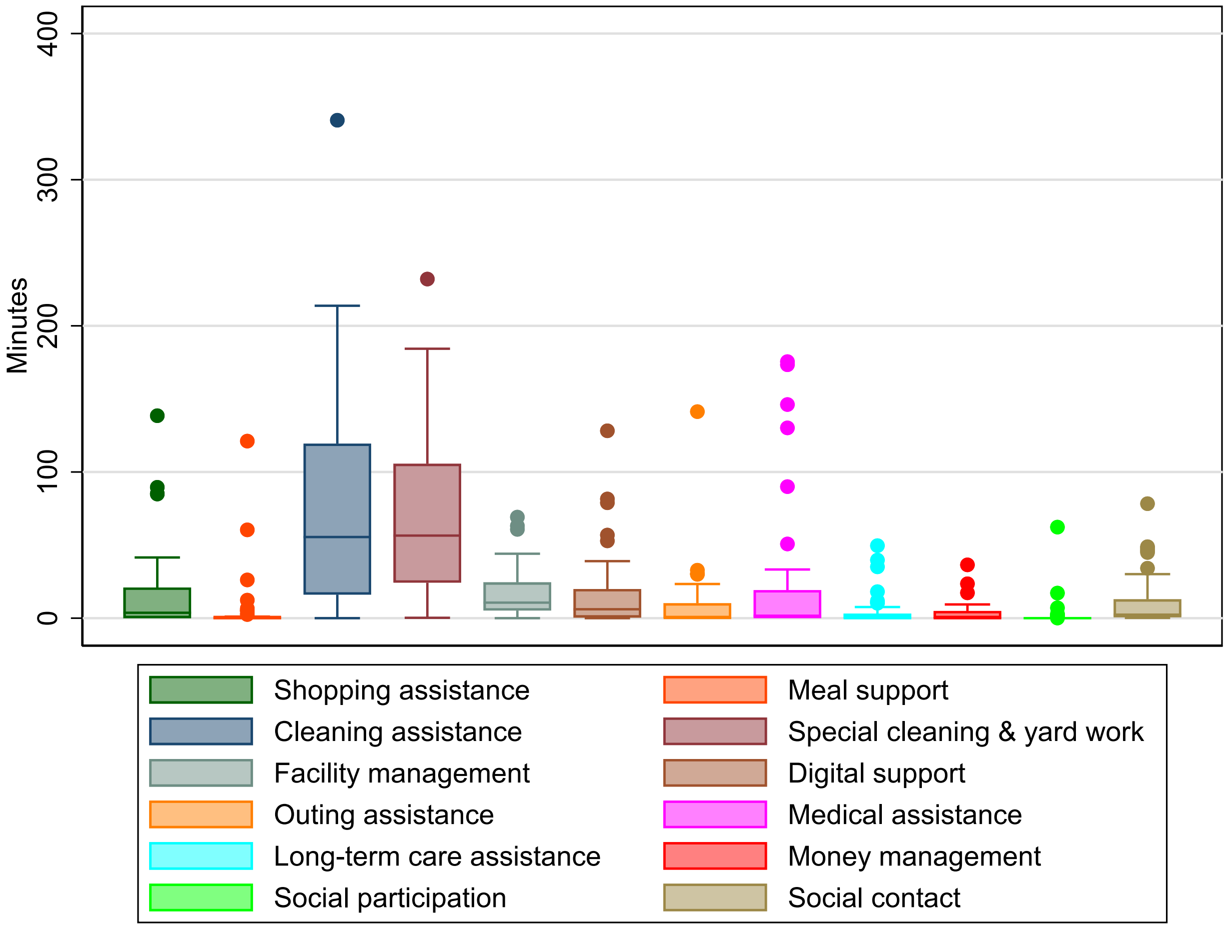

Figure S2 Monthly mean minutes of each service used during the first year of the contract. The monthly mean frequency was calculated by dividing the minutes of services used by the number of contracted days, multiplied by 30.

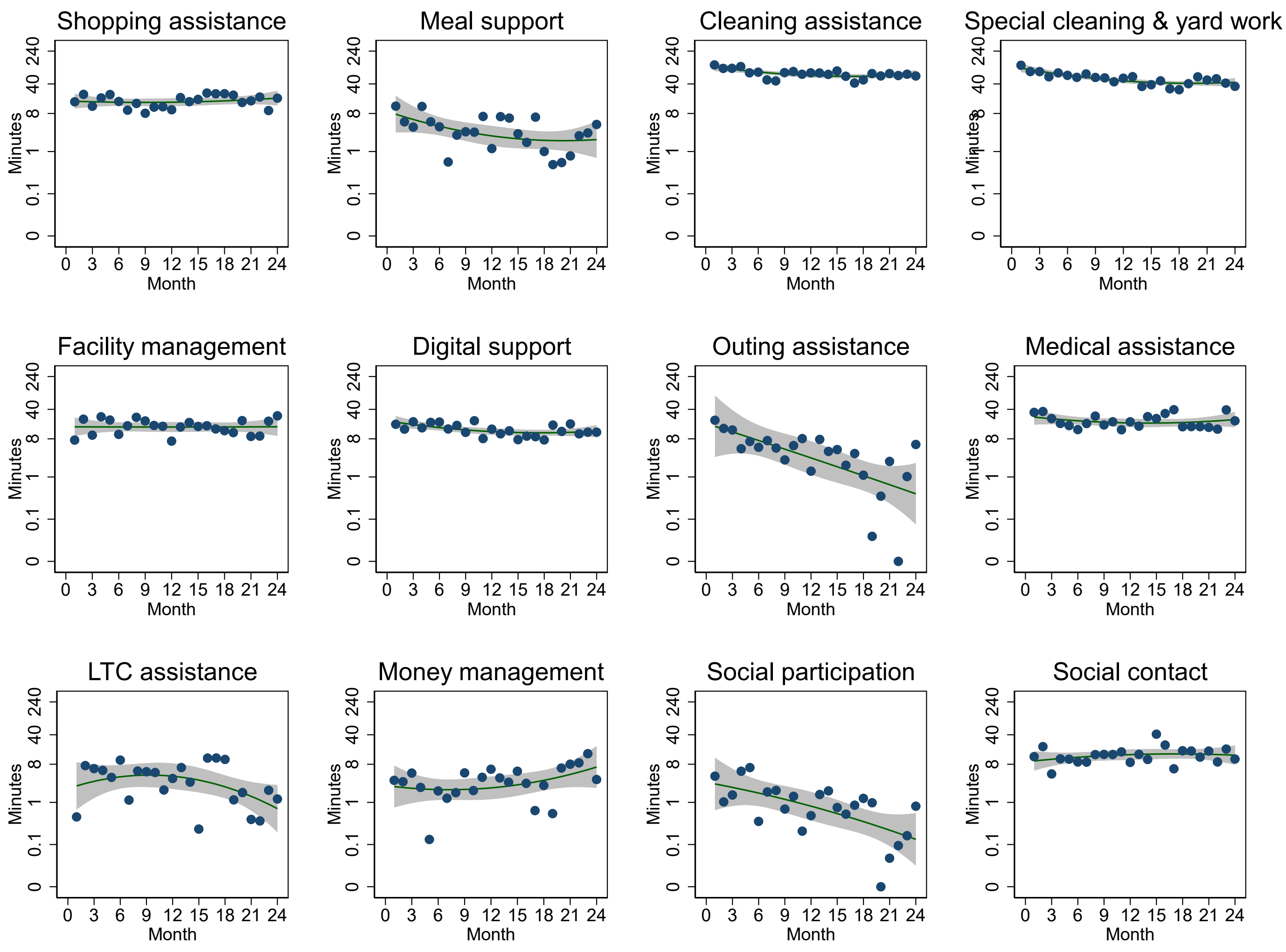

Figure S3 Trends in monthly mean minutes of each service used during the first two years of the contract. Scatterplots and quadratic prediction plots with 95% confidence intervals are shown.
